# Parainfectious Urticarial Rash in a Young Female with Epstein-Barr Virus Infection: A Case Report

**DOI:** 10.5811/cpcem.64029

**Published:** 2026-10-20

**Authors:** Huang XinYao Oliver, Andrea Yap, Dawn Toh, Lim Hoon Chin Steven

**Affiliations:** * Changi General Hospital, Department of Emergency Medicine, Singapore; † National University of Singapore, Yong Loo Lin School of Medicine, Singapore

**Keywords:** atypical urticaria, Epstein-Barr virus, urticarial vasculitis, case report

## Abstract

**Introduction:**

Urticarial rash is a common condition but can present atypically, making it difficult to clinically diagnose. Thorough assessment with awareness and exclusion of differentials is crucial for definitive treatment.

**Case Report:**

This is a case of a 23-year-old woman who presented with signs and symptoms suggestive of streptococcal pharyngitis and was treated with clarithromycin by primary care. Two weeks later, the patient was referred to the emergency department for malaise and an acute, rapidly progressing pruritic rash. Further evaluation revealed an Epstein-Barr virus (EBV) infection.

**Conclusion:**

This case highlights the differential diagnoses and diagnostic reasoning required for a patient with a rash and concurrent respiratory symptoms. Differential considerations include viral exanthem, delayed drug-induced reaction, EBV-associated antibiotic rash, scarlet fever, and urticarial vasculitis. Early identification of atypical urticarial lesions and the exclusion of clinical mimics are vital to guide appropriate therapy and optimize patient outcomes.

## INTRODUCTION

Urticaria is a common dermatological presentation characterized by transient, pruritic wheals that typically resolve within 24 hours and follow a benign, self-limiting course. It arises from a wide range of triggers, including infections, medications, and immune-mediated processes. Given the varied etiologies of acute eruptions, refining the differential diagnosis can be challenging, particularly when the presentation includes atypical features. We report the case of a young woman who developed an urticarial eruption in the setting of Epstein-Barr virus (EBV) infection, characterized by purpuric, prolonged lesions and prominent systemic symptoms. This case illustrates the diagnostic reasoning required to distinguish atypical urticaria from its differential diagnoses and highlights the systematic exclusion process necessary to optimize patient outcomes.

## CASE REPORT

A 23-year-old immunocompetent woman presented to the emergency department with prostration and a two-day history of rapidly progressing pruritic rash that started on her proximal thighs before becoming generalized, with the greatest distribution over her limbs and back.

She had a recent two-week history of fever, cough, runny nose, and sore throat that was treated by her family physician with a five-day course of clarithromycin (500 mg twice daily) due to presumed streptococcal pharyngitis (no specific testing). It was chosen due to her known allergy to amoxicillin-clavulanate potassium. The antibiotic course was completed three days before onset of her rash. She had also used two traditional Chinese medicines: watermelon powder for her oral ulcer three days prior to onset of rash; and pearl powder for sore throat for three days, which was discontinued on the same day that the rash appeared.

There was no history suggesting autoimmune disease, including recurrent ulcers, sicca symptoms, Raynaud. phenomenon or joint pain, and no family history of autoimmune disorders. There were no clinical features indicating rheumatic heart disease or glomerulonephritis as complications of scarlet fever. Previously, an allergic reaction to amoxicillin-clavulanate potassium resulted in an urticarial rash on both lower extremities.

On physical examination, there were erythematous urticated blanchable plaques over the inferior chin, thighs, shin, calves, and a few scattered on the abdomen, back, arms, hands, and soles. Purpuric plaques were noted on her calves (Image 1).

Oral cavity examination revealed bilateral erythematous tonsils without strawberry tongue or cervical lymphadenopathy. A small lower inner lip mucosal ulcer was noted. There were no target lesions, pustules, atypical targets, or mucocutaneous findings to suggest erythema multiforme, Stevens-Johnson syndrome, or other severe drug eruptions. Cardiovascular, renal, and joint examinations were unremarkable. Due to significant malaise, generalized weakness, and the purpuric feature of the rash, the patient was admitted for treatment and workup of systemic and autoimmune disorders.

Laboratory results revealed the following: microcytic hypochromic anemia with hemoglobin 9.9 g/dL (reference range, 12–16 g/dL); ferritin, 177 μg/L (7.6–179 μg/L), with further testing revealing alpha thalassemia; white blood cell count, 3.7 × 10^9^/L) (4×10^9^ –10×10^9^/L); C-reactive protein, 11 mg/L (0.2–9.1 mg/L); and erythrocyte sedimentation rate, 52 mm/h (3–15 mm/h). An autoimmune panel was negative for antinuclear antibodies, anti–double-stranded DNA antibodies, and extractable nuclear antigens; complement levels were normal. An infectious panel was positive for EBV by polymerase chain reaction but negative for antistreptolysin O, herpes simplex virus, and dengue serologies.

*CPC-EM Capsule*

What do we already know about this clinical entity?

*Urticaria is a common but usually self-limiting condition that arises from triggers such as infections, medications, and immune-mediated processes.*

What makes this presentation of disease reportable?

*Urticarial eruption occurred in a patient with Epstein-Barr virus infection who had atypical features of purpura, prolonged lesions, and prominent systemic symptoms.*

What is the major learning point?

*Urticaria with red flags, such as duration >24 hours, purpura and systemic symptoms, warrant a thorough workup.*

How might this improve emergency medicine practice?

*A structured differential for atypical urticaria helps emergency physicians exclude dangerous conditions, guiding workup and appropriate symptomatic care.*

The overall clinical presentation was consistent with a parainfectious urticarial eruption in the setting of EBV infection. The patient was treated twice daily with oral fexofenadine (180 mg), mometasone cream, and aqueous cream. Following two days of observation, her lesions improved, and she was discharged in stable condition while skin biopsy reports were pending. A subsequent punch biopsy of the purpuric lesion on the right calf revealed spongiotic dermatitis with no evidence of vasculitis, dysplasia, or malignancy.

At a two-week dermatology clinic follow-up, the lesions had resolved with minimal postinflammatory hyperpigmentation. Direct immunofluorescence (DIF) results were pending at that time, but the tissue specimen subsequently demonstrated faint, fine granular deposits of IgM and bright granular deposits of C3 within the walls of the upper dermal vessels. Although these findings suggested vasculitis, clinical and histopathological correlation was required. During the eight-week follow-up, testing for antineutrophil cytoplasmic antibodies was proposed to the patient; however, she declined further investigation because she remained systemically well without rash recurrence.

A young woman with a two-week history of upper respiratory tract infection symptoms treated with antibiotics presented with urticaria mixed with purpura on the limbs, associated with severe malaise and prostration. One differential diagnosis considered for the urticaria was a viral exanthem, given that the pruritic eruption followed an upper respiratory tract infection. Viral exanthems, however, generally present as maculopapular or morbilliform eruptions (Image 2). Urticarial eruptions involving transient, pruritic wheals are not a classic or common manifestation of a viral exanthem, although EBV has been uncommonly linked to urticarial presentations.^1^,^2^

Another differential diagnosis is a delayed drug eruption, supported by the patient’s recent completion of an antibiotic course. Although the patient had a documented history of an allergic reaction (urticaria) to amoxicillin-clavulanate potassium, amoxicillin is a penicillin combined with a β-lactamase inhibitor, whereas clarithromycin is a macrolide. There is no known cross-reactivity between these drug classes. However, the possibility of an independent, unconfirmed drug hypersensitivity had to be considered, necessitating a thorough medication history.

Alternatively, this could have represented an EBV-associated antibiotic rash. Patients with suspected streptococcal pharyngitis who receive antibiotics and subsequently develop an eruption are classically considered to have underlying EBV infection. The most commonly proposed mechanism of antibiotic-induced skin reactions in the setting of infectious mononucleosis is that the underlying viral infection triggers antibiotic hypersensitivity secondary to immune alterations, rather than a true, persistent drug allergy. This virus-mediated immune alteration typically leads to a generalized, delayed hypersensitivity response involving the palms and soles, especially after exposure to aminopenicillins such as amoxicillin (Image 3). However, because this patient received clarithromycin—typically considered a safe alternative—this etiology was less probable. Furthermore, although this reaction was historically considered pathognomonic for EBV infection, with an incidence of 80% to100%, recent studies suggest a much lower incidence of 15% to 33%.^3^

The referring family physician had also suspected scarlet fever, which is characterized by a sandpaper-like exanthem and a strawberry tongue (Image 4). This can be supported diagnostically by detecting elevated antistreptolysin O titer indicating recent group A streptococcal infection. Untreated scarlet fever risks systemic complications such as rheumatic heart disease and glomerulonephritis.^4^ In view of the severity of such complications, suspected scarlet fever should always be worked up and, if positive, treated with an appropriate antibiotic course. It was unlikely in this case because the patient’s age was above the usual demographic (5–15 years old), and her symptoms were not pathognomonic of the condition, with a different rash morphology and no scarlet tongue. Nevertheless, inpatient testing revealed normal antistreptolysin O titers.

While the above entities account for the common causes of such a rash, several atypical features in this patient’s presentation—lasting greater than 24 hours, co-existing with purpura, absence of angioedema and presence of systemic symptoms (fever, malaise)—suggested an alternative pathology. Such features raise the suspicion of urticarial vasculitis, a rare entity where type 3 immune complex-mediated inflammation produces urticarial lesions with purpura rather than wheals. This disease is largely idiopathic, though it has been linked to connective tissue diseases, hematologic diseases, and autoinflammatory syndromes.^5^ Our patient’s purpuric-looking lesions raised the suspicion of vasculitis and demanded a more thorough evaluation.

Studies have shown that EBV can be associated with vasculitis although it is considered rare.^6^–^9^ There have been case reports of EBV being implicated as a trigger of immune dysregulation in hypocomplementemic urticarial vasculitis (HUV). Tsai et al^10^ described a case of acute respiratory distress syndrome in a patient with HUV, where EBV was detected, and Berggren et al reported persistent EBV-related antigens in a patient with HUV.^11^ The proposed mechanism lies in molecular mimicry: anti-C1q auto-antibodies that cross-react with an EBV-derived nuclear antigen.^12^

The patient’s purpuric lesions therefore prompted a concern for vasculitic processes. Recognition of such cases is essential because early identification of an underlying systemic or autoimmune trigger can alter management.

The punch biopsy of the purpuric right-calf lesion showed spongiotic dermatitis with no convincing features of vasculitis, while the DIF of biopsy tissue showed faint, fine granular deposits of IgM and bright granular deposits of C3 seen within the walls of the upper dermal vessels, suggestive of vasculitis. The incongruence between routine histology and DIF may reflect the timing of the biopsy, given that classic leukocytoclastic changes can resolve rapidly as the acute inflammatory change subsides, leaving only residual immune complex deposition detectable on DIF.

For our patient, the short duration of rash and spontaneous resolution suggests a subtle, self-limiting parainfectious vasculitis, consistent with a transient type III immune complex-mediated reaction that did not cause significant longstanding histological changes.

## CONCLUSION

This report describes a patient with an EBV-associated parainfectious urticarial eruption who underwent detailed blood and histopathological evaluation. We systematically analyzed differential diagnoses, focusing on the exclusion of *Streptococcus* infection to guide rheumatic fever prevention measures. Prompt identification of atypical urticaria presentations and thorough diagnostic workups are vital to ensuring accurate clinical management and favorable patient outcomes.

## Figures and Tables

**Image 1:**
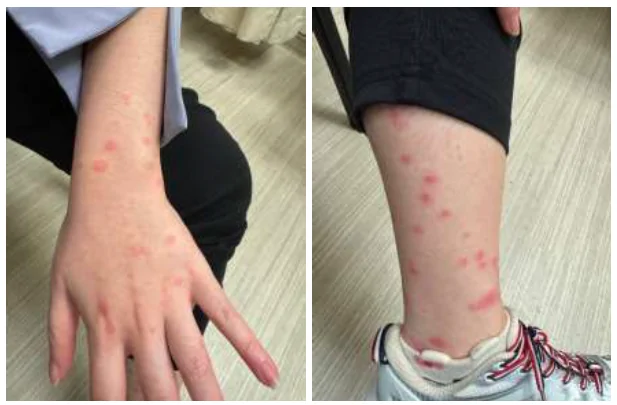
A) Dorsum of hand. B) An urticarial and purpuric eruption on lower extremities in a patient with Epstein-Barr virus infection.

**Image 2:**
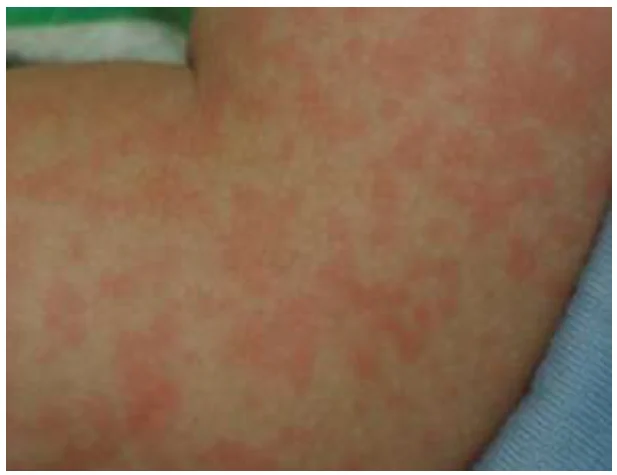
Characteristically maculopapular or morbilliform viral exanthem rash. Reprinted from Rehmus et al^13^ under the terms of the Creative Commons Attribution-NonCommercial-NoDerivatives 4.0 International (CC BY-NC-ND 4.0) License.

**Image 3:**
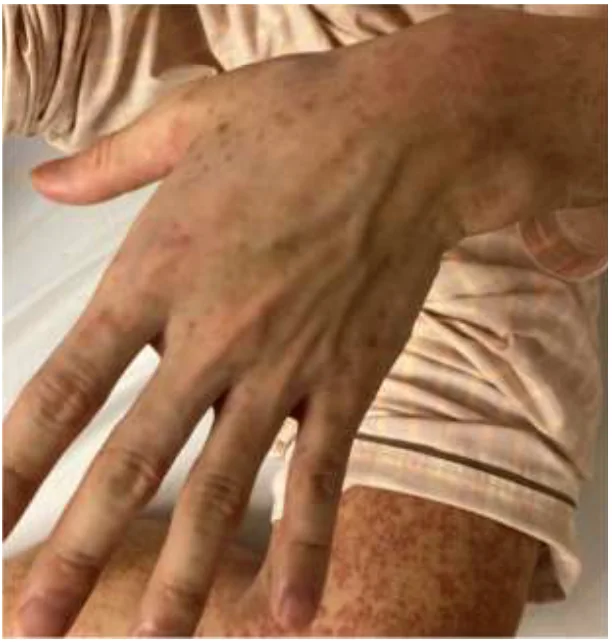
Erythematous, maculopapular eruption on the hand of a patient with an Epstein-Barr virus infection. Reprinted from Akiyama et al^14^ under the terms of the Creative Commons Attribution-NonCommercial-NoDerivatives 4.0 International (CC BY-NC-ND 4.0).

**Image 4:**
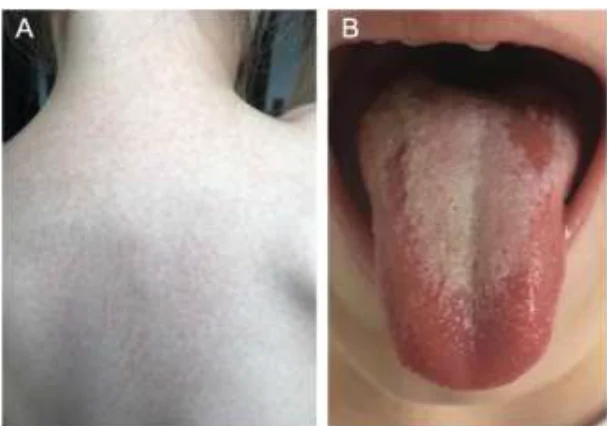
(A) Sandpaper-like exanthem on the back and (B) strawberry tongue in a 4-year-old patient with scarlet fever. Source: Ślebioda et al.^15^ This image is licensed under CC BY 3.0.
